# Atypical Within-Session Motor Procedural Learning after Traumatic Brain Injury but Well-Preserved Between-Session Procedural Memory Consolidation

**DOI:** 10.3389/fnhum.2018.00010

**Published:** 2018-01-30

**Authors:** Maria Korman, Sharon Shaklai, Keren Cisamariu, Carmit Gal, Rinatia Maaravi-Hesseg, Ishay Levy, Ofer Keren, Avi Karni, Yaron Sacher

**Affiliations:** ^1^Edmond. J. Safra Brain Research Center for the Study of Learning Disabilities, University of Haifa, Haifa, Israel; ^2^Loewenstein Rehabilitation Hospital, Ra'anana, Israel; ^3^Sackler Medical Faculty, Tel-Aviv University, Tel Aviv, Israel; ^4^Sagol Department of Neurobiology, Brain–Behavior Research Center, University of Haifa, Haifa, Israel; ^5^Sheba Medical Center, Ramat Gan, Israel

**Keywords:** procedural learning, motor sequence, atypical consolidation, training schedule, long-term memory, fatigue, TBI, memory deficits

## Abstract

Using the finger-to-thumb opposition sequence (FOS) learning task, we characterized motor skill learning in sub-acute patients hospitalized for rehabilitation following traumatic brain injury (TBI). Ten patients (Trained TBI) and 11 healthy participants (Trained Healthy) were trained using a multi-session protocol: a single session was afforded in the first week of the study, and four daily sessions were afforded during the second week. Intensity of practice was adapted to patients. Performance speed and accuracy were tested before and after each session. Retention was tested 1 month later. Ten patients (Control TBI) had no FOS training and were tested only at the beginning and the end of the 6 week period. Although baseline performance on the FOS was very slow, all three phases of skill learning found in healthy adults (acquisition, between-session consolidation gains, and long-term retention) could be identified in patients with TBI. However, their time-course of learning was atypical. The Trained TBI group improved in speed about double the spontaneous improvements observed in the Control TBI group, with no speed-accuracy tradeoff. Normalized to their initial performance on the FOS, the gains accrued by the Trained TBI group after a first training were comparable to those accrued by healthy adults. Only during the second week with daily training, the rate of improvement of the Trained TBI group lagged behind that of the Trained Healthy group, due to increasing within-sessions losses in performance speed; no such losses were found in healthy participants. The Functional Independence Measure scores at the start of the study correlated with the total gains attained at the end of the study; no correlations were found with severity of injury or explicit memory impairments. Despite within-sessions losses in performance, which we propose reflect cognitive fatigue, training resulted in robust overall learning and long-term retention in patients with moderate-severe TBI. Given that the gains in performance evolved mainly between sessions, as delayed, offline, gains, our results suggest that memory consolidation processes can be effectively engaged in patients with TBI. However, practice protocols and schedules may need to be optimized to better engage the potential for long-term plasticity in these patients.

## Introduction

Traumatic brain injury (TBI) is a leading cause of severe disabilities and handicap in individuals under the age of 45 in industrialized countries(Hillier et al., 1997; Tagliaferri et al., 2006). Whether these patients maintain the capacity to acquire and consolidate novel skills into long-term memory is of critical importance for the establishment of effective rehabilitation protocols.

### Memory deficits in TBI

Extensive memory deficits are one of the most common cognitive impairments leading to severe disability post TBI (Vakil, 2005). Multiple memory modalities can be affected. While explicit memory impairment is well-documented (Zec et al., 2001), less is known about procedural (“how to” knowledge) learning and memory deficits in patients with TBI, with disagreement about whether these occur and to what extent (Vakil, 2005). Even less is known about the time-course of multi-session training in TBI. Normal learning rates were observed by Nissley et al. (Nissley and Schmitter-Edgecombe, 2002) in perceptual learning task and Schmitter-Edgecombe et al. (Schmitter-Edgecombe and Rogers, 1997; Schmitter-Edgecombe and Beglinger, 2001) who examined the acquisition of skill in visual search and semantic-category memory search tasks. McDowall and Martin (1996) demonstrated intact acquisition gains and learning retention over a 20-min delay in practicing serial reaction time task. In contrast, Vakil et al., demonstrated that both explicit and implicit SRT memory measures are deficient in patients with TBI (Vakil et al., 2002). Additional study, using predictive saccade procedural learning paradigm, also showed significant deficits in learning gains in patients with TBI compared to matched healthy controls (Kohl et al., 2009). Altogether, current literature suggests that while task performance is impaired in patients with TBI, some procedural learning abilities may be relatively preserved (Vakil and Lev-Ran Galon, 2014).

Brain plasticity, the basis for skill learning, is a multi-phase and highly selective process, in which synaptic and cellular changes occur at neural networks initially engaged during salient experiences (Korman et al., 2003). The course of procedural learning in healthy individuals is well-documented in the finger opposition sequence (FOS) learning paradigm, the task used in the current study, with three distinguishable phases described (Karni et al., 1995, 1998; Korman et al., 2003; Walker, 2005): (i) Acquisition phase- fast within-session learning followed by a saturation phase with no additional improvement in performance despite continued practice. (ii) Consolidation phase-a latent phase lasting several hours, wherein sensitivity to interference decreases and additional, delayed “offline” gains emerge. Post-training affordance of conflicting tasks and/or poor sleep can hamper the course of learning a new motor sequence, by interacting selectively with the consolidation processes (Brashers-Krug et al., 1996; Korman et al., 2007; Lohse et al., 2014; Albouy et al., 2016; Friedman and Korman, 2016). The offline gains in performance presumably indicate the successful completion of procedural memory consolidation processes, commenced by the practice session but requiring additional time, including time in sleep, to evolve (Diekelmann et al., 2009; Borragán et al., 2015). (iii) Long-term retention phase - an extended skill generation phase contingent on multi-session training, wherein skill continues to improve both quantitatively and qualitatively, mainly through incremental between-session gains, with very robust long-term retention. Human imaging studies indicate that cortical, and sub-cortical, representations of the learned movement sequence are modified in all three phases, with structural level plasticity underlying the latter 2 phases (Albouy et al., 2016).

### The current study

Training protocols used in research of motor memory afford intensive training sessions, as the number of task repetitions is critical in determining the time-course of skill learning (Hauptmann et al., 2005; Wilhelm et al., 2012). However, such protocols may be suboptimal for individuals after TBI. One problem, however, that needs to be addressed in the transition from laboratory protocols of skill training that have been found to be appropriate for young healthy individuals, to patient groups, is that patients find these protocols too demanding. Often patients can comply only with less intensive training sessions. Individuals after a TBI, suffer from severe fatigue or mental exhaustion that interferes with activities of daily living (Dobryakova et al., 2013; Johansson et al., 2014), and pilot studies suggested that standard FOS training protocols need to be slowed and shortened for patients after TBI. Executive cognitive functions considered as a key factor in motor control and its deficits are associated with diminished muscle and proprioceptive abilities (Abd-Elfattah et al., 2015). Recently, a paradoxical, facilitating impact of cognitive fatigue on implicit procedural motor sequence learning, was found in healthy young adults (Borragán et al., 2016). The authors proposed that “facilitated learning in the high-level fatigue condition stems from a reduction in the cognitive resources devoted to cognitive control processes that normally oppose automatic procedural acquisition mechanisms”(Borragán et al., 2016). In explicit learning, where cognitive engagement is inherent, performance and learning is impaired when cognitive fatigue is imposed (Filoteo et al., 2010).

The aim of the current study was to evaluate motor skill acquisition abilities, and specifically procedural memory consolidation processes, in patients in the sub-acute phase of recovering from moderate-severe TBI, utilizing the FOS learning paradigm with reduced number of sequence repetitions per practice session, relative to the standard protocol (100 vs. 160 repetitions per training). A further aim was to test for a relationship between the severity of the injury, measures of cognitive and functional impairment, and skill learning abilities in these individuals. We hypothesized that FOS performance and learning ability will correlate with the magnitude of the functional impairment (measured using Functional Independence Measure, FIM; Seel et al., 2007) but not with the upper limb motor assessment scores (Fugl–Meyer; Feys et al., 2000) and the Manual Function Test, MFT; Michimata et al., 2008) and the cognitive assessments (Behavioral Assessment the Dysexecutive Syndrome, BADS; Engel-Yeger et al., 2009), the Rivermead Behavioral Memory Test, RBMT; Wiseman et al., 2000) due to their low variability and some missing data in the current sample of patients. FIM measure was previously suggested as a possible predictor of performance and training outcome (Shelton et al., 2001) in post-stroke rehabilitation.

## Methods

### Participants

Twenty sub-acute TBI participants hospitalized for rehabilitation were studied (Table 1). All were at least 2 months post injury and suffered non-penetrating moderate to severe TBI. All participants had to be able to perform all 4 finger opposition movements with the to-be-trained hand and to follow task instructions. Exclusion criteria were: previous neurologic, or psychiatric disease, clinical depression, direct neurologic or orthopedic trauma to the upper limb as well as severe pain that limited finger and wrist movements. Musicians or professional typists were excluded to avoid subjects with previous expertise on finger movement sequences.

**Table 1A:** Trained TBI group. Patients' characteristics.

**Patient No**.	**Age category**	**Time from injury (d)**	**Years of schooling**	**Beck scale**	**GCS**	**FIM**		**MFT**	**FUGEL MEYER**	**LOTCA impairments**	**BADS**	**RBMT**	**CT**
						**Admission**	**Study entrance**						
1	18–30	210	12	1	3	41	62	25	60	VM^@^ Reasoning	Impaired	7	DAI
2	30–40	94	12	15	9–12	77	87	32	60	Reasoning	16	21	Bilateral Frontal ContusionEDH^+^
3	30–40	153	12	8	<8	38	58	12	53	VM^@^, Praxis	Impaired	16	EDH^+^, SDH
4	30–40	73	12	15	7	40	101	32	59	Praxis	15	19	DAI
5	40–50	173	16	3	7	42	64	32	60	Orientation VM^@^ Attention	Impaired	4	DAI, SAH
6	30–40	90	12	5	7	23	23	31	40	Normal	Impaired	18	DAI
7	30–40	261	16	7	<8	32	107	26	60	VM^@^ Attention	18	22	DAI
8	30–40	80	16	13	<8	32	32	NA	60	Reasoning	NA	20	EDH^+^, SDH
9	18–30	69	18	9	<8	98	111	NA	60	Praxis	NA	NA	Rt. Temporal Contusion
10	18–30	66	12	7	9	110	116	NA	60	Normal	17	13	SDH, SAH
Mean	30	126.9	13.8	8.3		53.3	66.1	27.1	57.2			15.5	
SD	6.8	68.5	2.4	4.8		30.3	36.4	7.3	6.4			6.3	

**Table 1B:** Control TBI group. Patients' characteristics.

**Patient No**.	**Age category**	**Time from injury (d)**	**Years of schooling**	**Beck scale**	**GCS**	**FIM**		**MFT**	**FUGEL MEYER**	**LOTCA impairments**	**BADS**	**RBMT**	**CT**
						**Admission**	**Study entrance**						
1	40–50	113	11	9	6	49	87	32	60	Reasoning	Impaired	15	DAI + SAH contusion, in pons
2	30–40	130	12	13	3	75	118	32	59	Reasoning	18	22	DAI
3	40–50	151	12	11	3	28	90	22	54	Orientation Praxis VM^@^	18	19	SAH, SDH
4	18–30	111	12	1	5	43	73	29	60	VM^@^ Reasoning Attention	Impaired	14	SAH
5	30–40	110	9	5	<8	40	107	30	50	Orientation VM^@^ Reasoning	NA D/T aphasia	NA D/T aphasia	SAH
6	18–30	90	12	5	<8	45	121	32	60	VM^@^ Reasoning	20	20	SAH, SDH
7	18–30	121	12	3	<8	54	84	32	59	Reasoning	21	20	SDH, EDH, contusion
8	18–30	68	12	9	3	54	56	NA	54	Reasoning	21	17	Frontal contusion, edema
9	18–30	76	12	5	3	49	87	32	49	VM^@^	NA	20	DAI
10	18–30	214	12	NA	<8	31	91	NA	49	VM^@^ Reasoning Attention	NA	12	IVH, SAH
Mean	29.3	118.4	11.6	6.8		46.8	91.4	30.1	55.4			17.6	
SD	11.5	41.7	2.4	3.9		13.2	19.7	3.5	4.7			3.4	

*NA, Not Available; VM, Visio-Motor; DAI, Diffuse Axonal Injury; EDH, Epidural Hemorrhage; SDH, Subdural Hemorrhage; SAH, Subarachnoid Hemorrhage*.

Ten consecutive patients conforming with the inclusion criteria (seven males and three females) were included in the intervention group that afforded a multi-session training on a given sequence of finger movements (Trained TBI group), Seven of them were eligible for retesting a month later in order to assess retention. Another group of 10 consecutive patients (all males) were included in the control TBI group. All patients with TBI were right handed, except one in the control group. In the study group six patients had bilateral paresis and four patients had hemiparesis (two right, two left) and in the control group seven patients had double hemiparesis, two patients had right hemiparesis and one had left hemiparesis. In the Trained TBI group all patients executed the sequence with their best functioning limb: eight patients used their right hand and two used their left non-dominant hand. In the Control TBI group all three patients with unilateral hemiparesis preferred to execute the sequence with their paretic hand and among the double paretic four patients used their right hand and three used their left one. None had complaints of pain during the study period; however patient 6 (Tables 1A,B) from the Trained group rated his trained arm pain as VAS six at the beginning of the study and patients 2 and 9 rated their pain level on entering the study as 5 and 4, respectively. All patients were hospitalized in a rehabilitation department for the whole study period and were afforded the same rehabilitation program.

Severity of injury was assessed by the Glasgow Coma Scale (GCS) (Teasdale and Jennett, 1974), length of unconsciousness (LOC) or length of post-traumatic amnesia (PTA). Brain imagining findings were drawn from medical records. All participants underwent functional evaluation that included the following measures: Functional Independence Measure (FIM) (Seel et al., 2007) to assess activities of daily living (ADL) abilities, Fugl–Meyer (Feys et al., 2000) and the Manual Function Test (MFT) (Michimata et al., 2008) for upper limb motor assessment and the visual analog scale (VAS) for pain assessment. Cognitive evaluation was based on the Loewenstein Occupational Therapy Cognitive Assessment (LOTCA) (Katz et al., 1989) battery, Behavioral Assessment the Dysexecutive Syndrome (BADS) (Engel-Yeger et al., 2009), and the Rivermead Behavioral Memory Test (RBMT) (Wiseman et al., 2000). The Beck Depression Inventory Scale was used for emotional screening, where scores higher than 16 points were an exclusion criterion (Beck et al., 1988). The mean Beck Depression Inventory scores was 8.3 ± 4.8 (intervention) and 6.8 ± 3.2 (control), corresponding to minimal depressive symptoms). Details regarding patients' characteristics are presented in Table 1.

Eleven healthy female controls were enrolled in the Healthy Control group. All of them were right-handed and used their left, non-dominant hand, to execute the movements. Individuals with neurological, sleep, cardiovascular or musculoskeletal dysfunctions, individuals with learning disabilities, smokers (over 3 cigarettes a weak) and alcohol consumers (over 3 portions per week), individuals with extreme body mass index (17 < BMI < 29) and individuals with shifts work were excluded. Mean age (29 ± 6.8) and mean education years (13.8 ± 2.4) were matched to the Trained TBI group.

### Task and procedure

**Figure 1:**
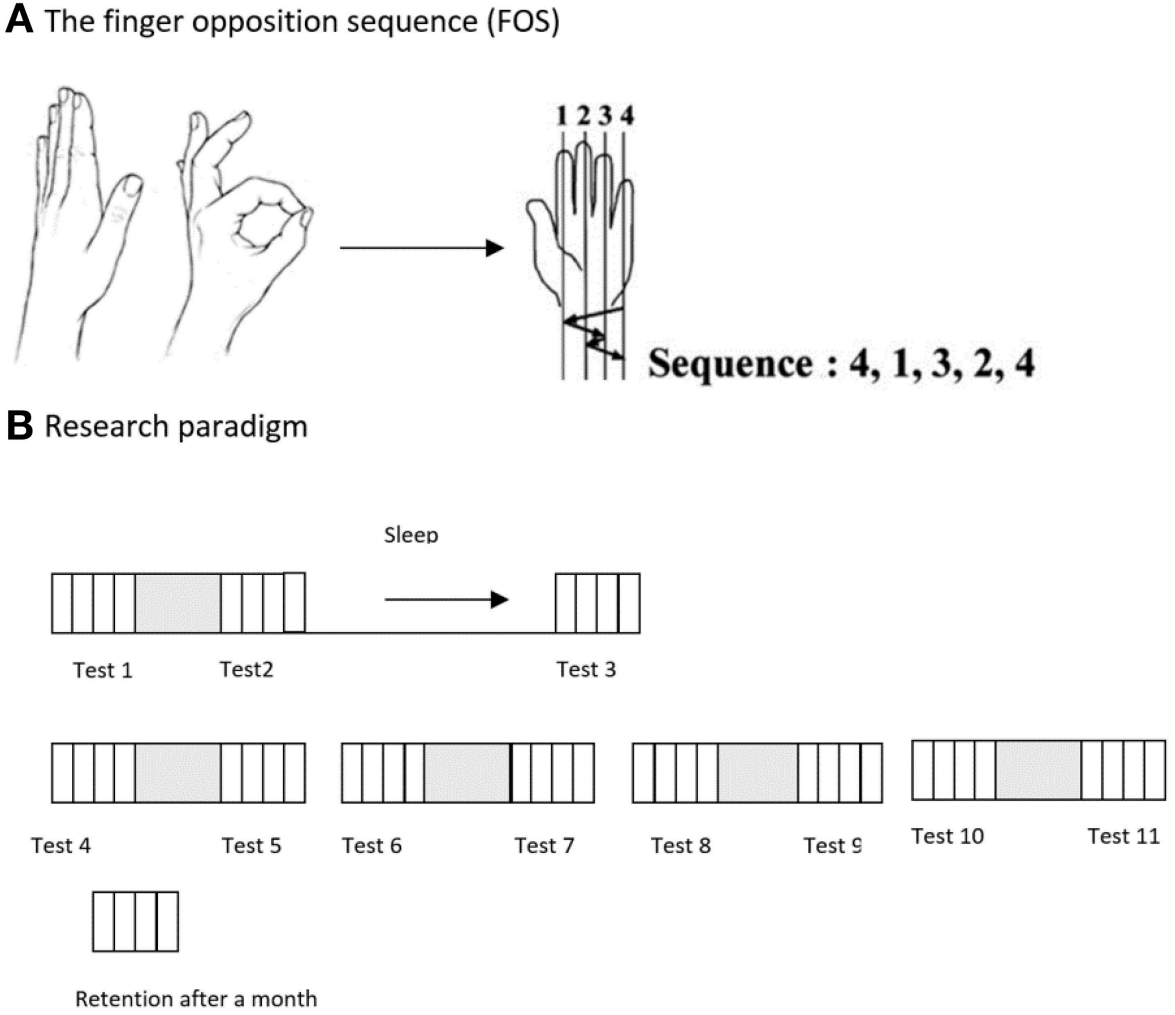
**(A)** The Finger Opposition Paradigm: participants were asked to perform, repeatedly, a sequence of finger opposition movements as quickly and accurately as possible. **(B)** The time-line of the study: on the 1st week of the study the examinees were tested 3 times, before practicing the sequence, after the practice session, and at 24 h post-training after a night's sleep (Tests 1, 2, and 3). On the 2nd week participants were tested 8 times, before and after each of the 4 consecutive daily training sessions (Tests 4–11). An additional retention test was carried out 4 weeks after the final training session. Each performance test comprised four blocks of 30 s continuous performance of the assigned FOS.

All subjects were shown and explicitly instructed a 5-element sequence of finger to thumb opposition movements (FOS) as previously described (21) numbering the fingers 1–4, with 1 designating the index finger and 4 the little finger: 4-1-3-2-4 (Figure 1A). The participants were instructed and repeatedly encouraged to oppose the fingers of the examined hand to the thumb in the given movements sequence “as quickly and accurately as possible” during tests. The participants performed the instructed movements in direct view (palm-facing) of a video camera, to allow recording of all finger movements. Participants were instructed to divert their gaze so that visual feedback was not afforded.

Participants of the Trained TBI and Trained Healthy groups were trained to perform a 5-element FOS using an intensive multi-session protocol: the sequence was practiced in a single session in the first week of the study, and daily during the second week. The training session began only after three consecutive correct sequences were executed by the trainee, indicating that the participant knew what movements were required to execute the sequence correctly. Each practice session was composed of 10 blocks of 10 repetitions of the sequence, each sequence cued at a comfortable rate (auditory, 0.28 Hz, allowing 3.5 s for the completion of each sequence). Thus, each practice session included altogether 100 repetitions of the FOS. The performance tests consisted of four intervals (blocks) of 30 s each, with clear auditory “start” and “stop” cues, during which the participants were asked to tap the sequence, repeatedly, as quickly and accurately as possible. Participants were instructed that if they become aware of committing an error they should continue with the task. Participants practiced the sequence in five training sessions during the 2-week intervention period. At the beginning of the first week, an initial session was afforded. Starting from the beginning of the second week, four additional consecutive daily practice sessions were performed. Eleven performance tests were performed during both weeks of the study, before and after each of the five practice sessions and by 24 h after the initial practice session (on week 1) and an additional test was run 4 weeks after the final practice session was completed for the participants of the Trained TBI group (retention test). No further training was given during the month between Test 11 and the retention test (Figure 1B).

Participants of the Control TBI group were tested twice on the performance of the sequence, at the beginning and end of a month period. No training was afforded to the control patients.

All tests and practice sessions were video-recorded and analyzed offline. The number of correct and incorrect executed sequences were scored for each test-block. Statistical analysis was carried out using the SPSS (Version 23) software; repeated measures ANOVA GLM, *t*-test and Pearson's correlation matrix were utilized. Analyses were performed separately for speed (mean of the number of correct sequences executed during the performance test-block) and for accuracy (mean of the number of incorrect sequences during the performance test-block) of performance. At each time-point, mean speed and accuracy performance scores across the four test blocks were calculated.

The study was approved by the Human Research Ethics Committee of the Lowenstein Rehabilitation Hospital and the Israeli Ministry of Health. All participants and their guardians gave written informed consent before inclusion.

## Results

### Demographic data

Table 2 summarizes the comparisons of key demographic and neurocognitive data for the experimental groups (Trained TBI, Control TBI) as well as demographic data for the Trained Healthy control participants. Note that the only significant difference found between the 2 TBI groups was for years of education. The actual difference between the means was 2 years (13.8 vs. 11.6); all participants had completed high-school.

**Table 2:** Independent samples, 2-tailed *t*-tests for demographic characteristics.

	**Trained TBI vs. Control TBI**			**Trained TBI vs. Control Healthy**			**Control TBI vs. Control Healthy**		
	**t**	**df**	**p**	**t**	**df**	**p**	**t**	**df**	**p**
Age	0.164	18	0.871	0.368	19	0.717	0.077	19	0.940
Years of schooling	2.694	18	**0.015**	−1.237	19	0.231	−4.778	19	**<0.001**
Time from injury (d)	0.335	18	0.742						
Beck scale	0.750	17	0.463						
FIM admission	0.621	18	0.542						
FIM study entrance	−1.929	18	0.070						
MFT	−1.031	13	0.321						
FUGEL MEYER	0.712	18	0.486						
RBMT	−1.311	17	0.207						

*Bold indicates significant values of p*.

### Behavioral data

There were no pre-training differences in terms of the number of correct sequences produced between the experimental TBI groups (means: 7.05 ± 2.69, 7.06 ± 3.81, Trained TBI, Control TBI, respectively; independent samples 2-tailed *t*-test, *p* = 0.996, *d* = 0.003). On average, the participants in both groups committed very few errors (means: 1.5 ± 1.3, 0.86 ± 0.65, Trained TBI, Control TBI, respectively; *p* = 0.09, *d* = 0.62).

#### Trained vs. un-trained patients with TBI

**Figure 2:**
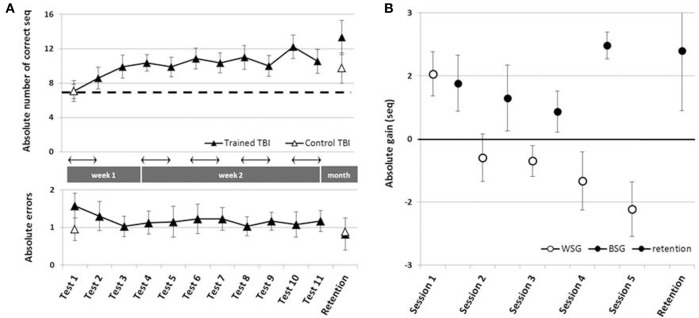
Time-course of improvement across multi-session training. Open triangles–Control TBI group, filled triangles–Trained TBI group. **(A)** Speed and accuracy of performance across the training and the retention periods. Upper panel–speed; group average of the absolute number of correct sequences at each of the 11 performance tests and the retention test. Arrow–Session. Lower panel–accuracy; group average of the number of incorrect sequences performed at each test. **(B)** Absolute within-session and between-sessions gains in performance speed: the within-session gains (WSG)-black circles, were defined as the difference between the absolute performance scores at tests before and after a given training (e.g., Test2-Test1, Test5-Test4, etc.). The between-session gains (BSG)-open circles, were defined as changes in performance scores between the successive tests of different days, known also as consolidation gains (e.g., Test3-Test2, Test6-Test5).

To address the main research questions, the time-course of performance changes as a function of multiple practice sessions afforded to the Trained TBI group, was analyzed. Training on the given sequence of movements, afforded in the five training sessions, resulted in robust gains in speed performance (Figure 2A, upper panel). Repeated measures ANOVA with 11 time points (Tests 1–11) spanning the whole intervention period showed that there was a significant improvement in performance speed over the 2 weeks of the study [*F*_(10, 90)_ = 5.677, *p* < 0.001, MSE = 57.82; η^2^ = 0.41], with participants able to tap, on average, an additional 5.18 ± 3.35 correct sequences at the beginning of the final training session (Test 10) above the number at the pre intervention, Test 1. Absolute accuracy was high (Figure 2A, lower panel). The number of errors produced showed no significant changes across the intervention period [Tests 1–11; *F*_(10, 80)_ = 0.356, *p* = 0.962 MSE = 1.09; η^2^ = 0.04]. Seven out of 10 participants of the Trained TBI group were available for testing for retention a month after the final training session. There was no evidence for forgetting [comparison of Test 10 and the retention test *F*_(1, 6)_ = 1.034, *p* = 0.348, MSE = 3.50; η = 0.15].

Positive values correspond to improvement; open circles - within-session gains (WSG) calculated as [mean post-training score - mean pre-training score for each Session(N)]. Note that WSG is positive only in the first session. Black circles - between-session gains (BSG), calculated as [mean pre-training score of the Session(N) - mean post-training score of the Session(N-1)]. Black triangle - retention gain (7 participants) calculated as [mean retention score - mean post-training score at Session(5)]. Bars–SE of the mean.

Next, we tested the possibility that spontaneous improvement in performance occurred in the Control TBI group between the pre-test and a test conducted 6 weeks later, the latter corresponding to the retention test administered to the Trained TBI group (Figure 2A). Repeated measures ANOVA with 2 time points (Test 1, retention) showed that there was a significant improvement in performance speed over the month period [*F*_(1, 9)_ = 12.84, *p* = 0.006, MSE = 36.45; η^2^ = 0.59], with additional 2.7 ± 2.26 correct sequences in the retention test block. This improvement was not at the expense of accuracy. The absolute number of errors produced was not increased (did not change) across the intervention period [*F*_(1, 9)_ = 0.10, *p* = 0.755, MSE = 0.28; η^2^ = 0.01].

The multi-session training protocol afforded to the Trained TBI group resulted in significantly larger gains in performance speed compared to the spontaneous gains attained over a similar time interval. A repeated measures ANOVA with 2 groups (Trained TBI, Control TBI) × 2 time points (Test 1, retention) showed a significant interaction of time-point × group [*F*_(1, 15)_ = 7.33, *p* = 0.016, MSE = 33.17; η^2^ = 0.33] reflecting the higher gains in performance attained by the Trained TBI group, although both groups improved across the study period [*F*_(1, 15)_ = 40.31, *p* < 0.001, MSE = 182.47; η^2^ = 0.73] (Figure 2A, upper panel, black, and open triangles).

As can be seen in Figure 2B, the behavior of the Trained TBI group during the 1st week of the study, i.e., during and following the 1st practice session was different from that observed on week 2. Only in the second week (during the 4 daily sessions) the patients' performance was characterized by a series of loses during the sessions and gains between-sessions, with the latter overriding the within-session losses. Therefore, additional analyses were run to explore this apparently differential behavior of the patients in the 1st vs. 2nd week of the study, focusing on two distinct time-windows (that have been previously explored in young healthy adults; Karni et al., 1995): the immediate and delayed effects of the first training session; the effects of training sessions 2–5 at week 2 and retention a month later.

**Figure 3:**
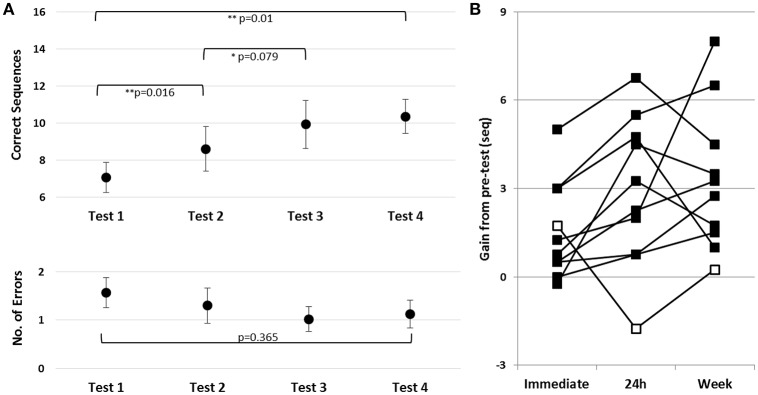
Effects of a single training session in the Trained TBI group. **(A)** Time-course of improvement in performance. Upper panel–speed; group average of the absolute number of correct sequences at each of the four trials of the performance tests (pre-training, post-, 24 h- and week- post-training). Lower panel–accuracy; Respective errors–group average of the number of incorrect sequences performed at each test. Bars–SE of the mean. **(B)** Absolute individual gains (Δ) relative to pre-training baseline performance, calculated as [Immediate = (Test 2–Test 1); 24 h = (Test 3–Test 1); Week = (Test 4–Test 1)]. Open square–case patient with a decrease in performance at Test 3 (negative gains) note recovery a week later, at Test 4.

**Figure 4:**
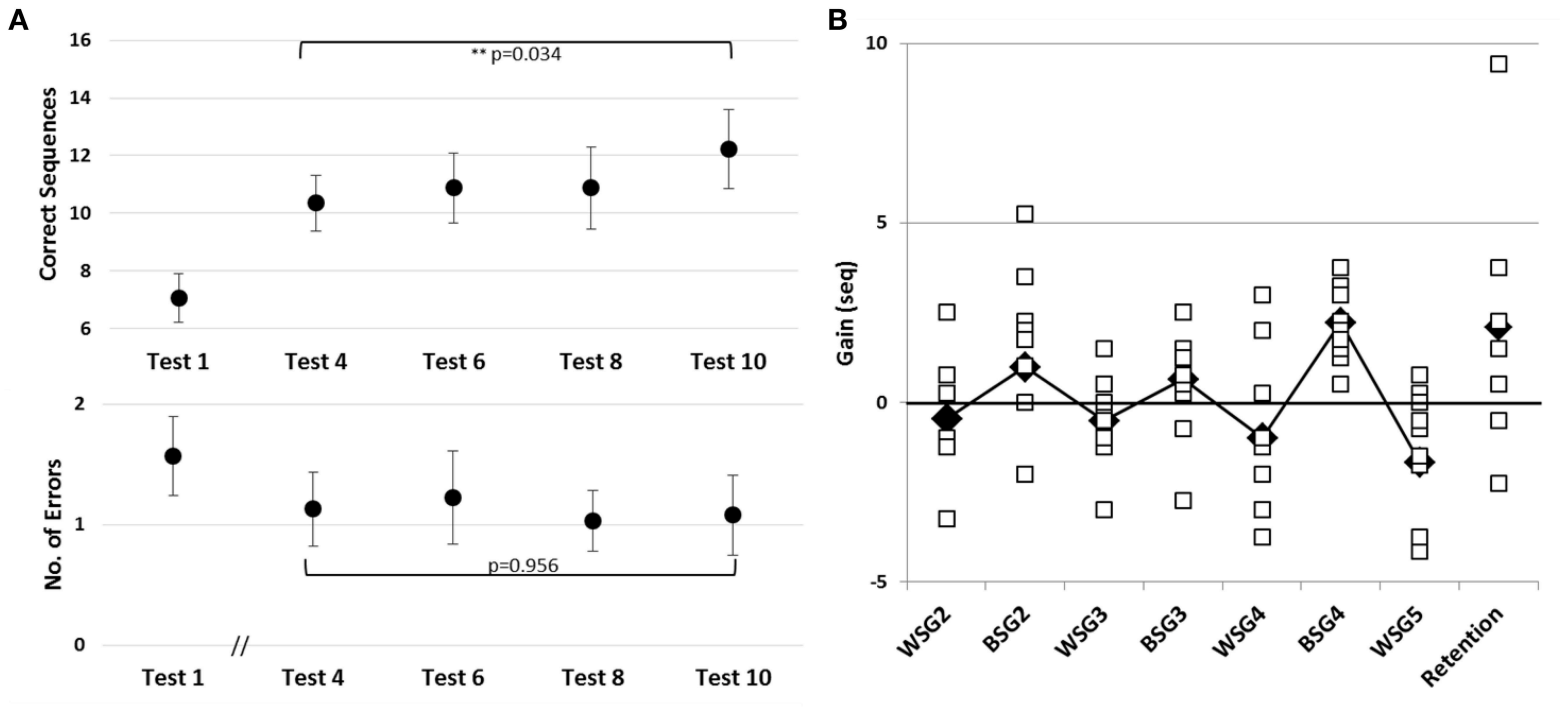
Learning across the four training sessions of the second week of intervention and performance at retention. **(A)** Pre-tests of Sessions 2–5 are shown. Performance at Session 1 (Test 1) is presented as a reference point for improvements during the second week (Tests 4, 6, 8, and 10). Upper and Lower panels–as in Figure 3. **(B)** Individual within-session gains, WSG 2-5, and between-session gains, BSG 2-4 are shown, calculated as in Figure 2B. Black diamonds–group means; Retention–mean and individual gains relative to Test 12 (*N* = 7).

To assess learning during and following the first training session, in Trained TBI group, a repeated measures ANOVA was run with the following time-points included: pre-training, post-training, 24 h post-training and the test immediately preceding practice session 2 (Tests 1–4, respectively). There was a robust improvement in speed [*F*_(3, 27)_ = 7.71; *p* = 0.01, MSE = 22.13; η^2^ = 0.46] with no costs in accuracy [*F*_(3, 27)_ = 1.091, *p* = 0.370, MSE = 0.58; η^2^ = 0.11] (Figure 3B, upper and lower panels, respectively). Pair-wise comparisons, with Bonferroni correction for multiple comparisons, showed a significant gain in performance speed within the session (Tests 1–2, *p* = 0.016, *d* = 0.46). The group's overnight improvement in performance was only marginally significant (Tests 2–3, *p* = 0.079, *d* = 0.32). However, examination of individual's gains revealed that overnight, additional gains were present in 9/10 participants (Figure 3B, black squares); one patient, in contrast, had a marked loss in performance at Test 3 (Figure 3B, open square). Excluding the atypical patient, robust overnight gains were apparent (Nine patients; Test 2–Test 3, *p* = 0.003, *d* = 0.44). Note, moreover, that the individual who failed to show the delayed gains by 24h post-training recovered the gains attained in the first training session after an additional interval of 5 days (Figure 4B, Test 4). Thus, all of the patients showed a tendency to express delayed, consolidation phase gains by Test 4 (prior to the 2nd training session) compared to the immediate post-training performance (Test 2) (two-tailed paired *t*-test, *p* = 0.067, *d* = 0.49).

Despite an overall improvement of performance, the second week of training resulted in a dissociation between the short term (immediate) and long-term benefits of each practice session (Figure 2B). The participants' performance at the end of most of the training sessions was significantly worse than their performance at the beginning of each training session, i.e., the within-session gains (WSG) accrued across the second week of the study period became negative (Figure 2B). Moreover, the deterioration of performance across the practice sessions became more pronounced in the latter sessions of week 2 [WSGs over 5 intervals, *F*_(4, 36)_ = 5.012; *p* = 0.003, MSE = 14.46; η^2^ = 0.36]. In contrast, the between-session gains (BSG) were consistently positive over the study period [BSGs over 4 intervals, *F*_(3, 27)_ = 2.075; *p* = 0.127, MSE = 4.61; η^2^ = 0.19] (Figure 2B).

**Figure 5:**
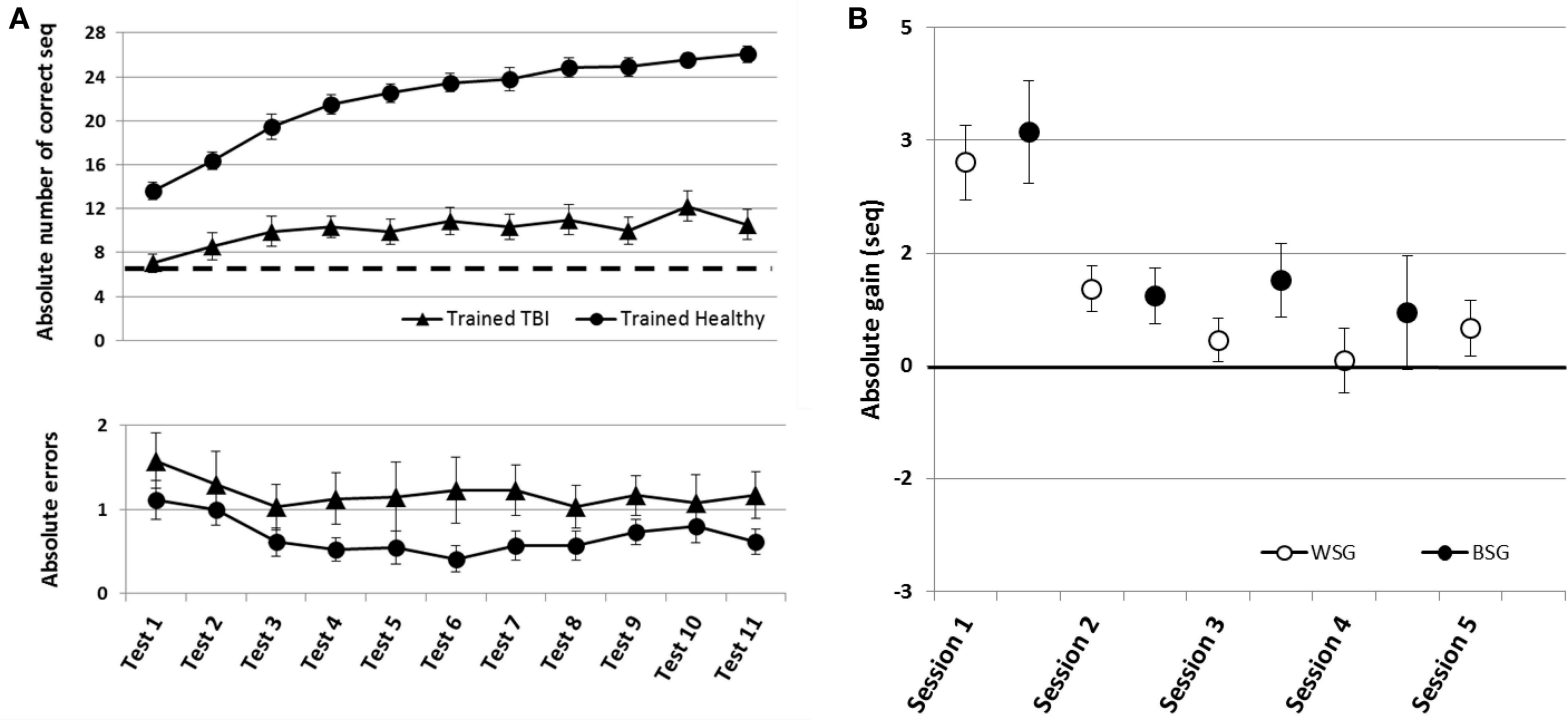
Time-course of improvement across the multi-session training period. Triangles–Trained TBI group, Circles–Trained Healthy group. **(A)** Speed and accuracy of performance. Upper panel–speed, group average of the absolute number of correct sequences at each of the 11 performance tests. Double arrow–Session. Lower panel–accuracy, group average of the number of incorrect sequences performed at each test. **(B)** Absolute within-session and between-sessions gains in performance speed of the Trained Healthy group, as in Figure 2.

To assess learning across the four additional training sessions afforded on the second week, irrespective of the within-session decreases in performance, only the pre-session scores of the consecutive training sessions (Tests 4, 6, 8, and 10) were compared. There was an average improvement by 3.30 ± 2.58 sequences that participants were able to tap at Test 10 compared to Test 4 [*F*_(3, 27)_ = 3.351; *p* = 0.034, MSE = 6.30; η^2^ = 0.27] indicating a robust overall increase in performance speed (Figure 4A, upper panel). However, pair-wise comparisons between these consecutive pre-scores were not significant; suggesting that starting from the second training session the improvement in speed occurred in slow incremental steps. There was no increase in the number of errors committed during the 2nd week pre-tests (Tests 4, 6, 8, and 10), with participants maintaining very high levels of accuracy throughout [Tests 4, 6, 8, and 10; *F*_(3, 27)_ = 0.105, *p* = 0.956, MSE = 0.073; η^2^ = 0.012] (Figure 5A, lower panel).

#### Trained patients with TBI vs. trained healthy participants

Healthy controls trained using the adapted-for-patients training protocol, improved robustly their performance, but the gains were expressed in a somewhat different time-course, specifically in the second week of the training protocol (Figure 5). The pre-training performance of the healthy controls was, as expected, significantly better compared to the patients with TBI in terms of the mean number of correct sequences (mean 13.63 ± 3.15; two-tailed *t*-test, Trained TBI, Trained Healthy, *p* < 0.001, *d* = 2.24) but not in the absolute number of errors (mean1.11 ± 1.12; two-tailed *t*-test, Trained TBI, Trained Healthy, *p* = 0.98, *d* = 0.32). Participants of the Trained Healthy group, as expected, showed robust improvement in performance speed [*F*_(10, 100)_ = 66.193; *p* < 0.001, MSE = 0.703.18; η^2^ = 0.87] across the training interval (Tests 1–11). Indeed, compared to their performance at Test 1 the healthy participants were able to tap on average an additional 12.43 ± 3.15 correct sequences in the final test block (Test 11) (Figure 5A, upper and lower panels). A repeated measures ANOVA with 2 groups (Trained TBI, Trained Healthy) and 11 time points (Test 1–11) as a within-subject factor, showed a significant group effect [*F*_(1, 19)_ = 84.69; *p* < 0.001, MSE = 32785.23; η^2^ = 0.817] reflecting the large advantage of the Trained Healthy participants in overall speed of performance, as well as an interaction of time-point × group, [*F*_(10, 190)_ = 14.880, *p* < 0.001, MSE = 173.04; η^2^ = 0.439]. This interaction reflected the fact that the gains in performance speed attained by the Trained Healthy group were not only higher but also followed a different time-course (Figure 5A, upper panel). The main difference in the time-course of skill learning between the two trained groups was related to the fact that the Trained Healthy group did not show negative within-session gains in any of the training sessions (Figure 5B compared to Figure 2B).

**Figure 6:**
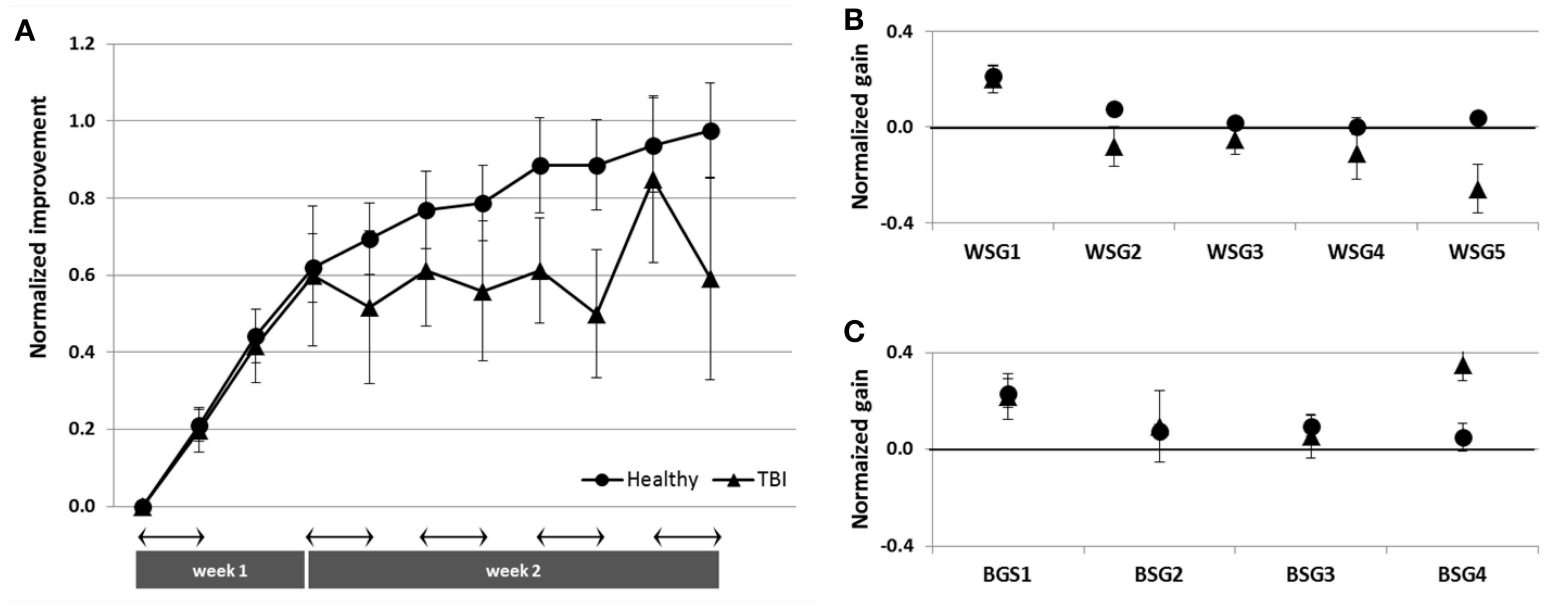
Normalized data for the Trained TBI (triangles) and the Trained Healthy (circles) groups. **(A)** Time-course of improvement across multi-session training. **(B)** Normalized within-session gains (WSGs) and **(C)** normalized between-session gains (BSGs).

To directly compare the time-courses of motor skill evolution in the TBI and the Healthy groups, normalized gains were calculated for each participant [(mean performance at Test (N) minus mean performance at Test 1) divided by performance at Test 1; Figure 2B]. During the first week, the normalized performance (i.e., expressed as normalized values vis-à-vis each participant's Test 1) of both trained groups were very similar (Figure 6A). A repeated measures ANOVA with 2 groups (Trained TBI, Trained Healthy) × 3 time points (Test 2–Test 4), showed no group effect [*F*_(1, 19)_ = 0.036, *p* = 0.851, MSE 0.006.81; η^2^ = 0.002], no interaction [*F*_(2, 38)_ = 0.003, *p* = 0.997, MSE < 0.001; η^2^ < 0.001] and significant time point effect [*F*_(2, 38)_ = 2.86, *p* < 0.001, MSE = 0.861; η^2^ = 0.398]. The emerging differential in the time-course of gaining skill was clearly reflected in the repeated measures ANOVA with normalized performance scores (2 groups, Trained TBI, Trained Healthy) × 7 time points (Test 4–Test 11) showed a significant interaction of time-point × group [*F*_(7, 133)_ = 2.323, *p* = 0.029, MSE = 0.089; η^2^ = 0.11] and significant time point effect [*F*_(7, 133)_ = 4.941, *p* < 0.001, MSE = 0.190; η^2^ = 0.206], suggesting different time-courses of skill learning. Normalized within-session and between session gains were calculated for each study participant. A repeated measures ANOVA with 2 groups (Trained TBI, Trained Healthy) × 5 within-session intervals (WGSs 1-5) was conducted using the normalized gain scores. There was a significant group effect [*F*_(1, 19)_ = 5.821; *p* = 0.026, MSE = 0.448; η^2^ = 0.24] and a significant interaction of WSG interval × group, [*F*_(4, 76)_ = 2.395, *p* = 0.051, MSE = 0.120; η^2^ = 0.19], suggesting differences in both the magnitude and the pattern of WSGs between groups of patients and healthy participants (Figure 6B). In both groups the WSGs gains showed significant decrease over the study period [*F*_(4, 76)_ = 9.944, *p* < 0.001, MSE = 0.299; η^2^ = 0.34]. In contrast, there were no significant differences between the groups in terms of the normalized between-session gains (BSGs) (Figure 6C). A repeated measures ANOVA [2 groups (Trained TBI, Trained Healthy) × 4 between-session intervals (BSGs 1-4)] using the normalized gain scores, showed no significant group effect [*F*_(1, 19)_ = 0.831, *p* = 0.373, MSE = 0.088; η^2^ = 0.042]. There was a marginally significant interaction of BSG interval × group, [*F*_(3, 57)_ = 2.395, *p* = 0.067, MSE = 0.130; η^2^ = 0.12], reflecting the higher between-session gains of the Trained TBI group in the final interval. There was a marginally significant tendency for the BSGs to decrease over the study period [*F*_(3, 57)_ = 2.404, *p* = 0.077, MSE = 0.124; η^2^ = 0.11].

**Figure 7:**
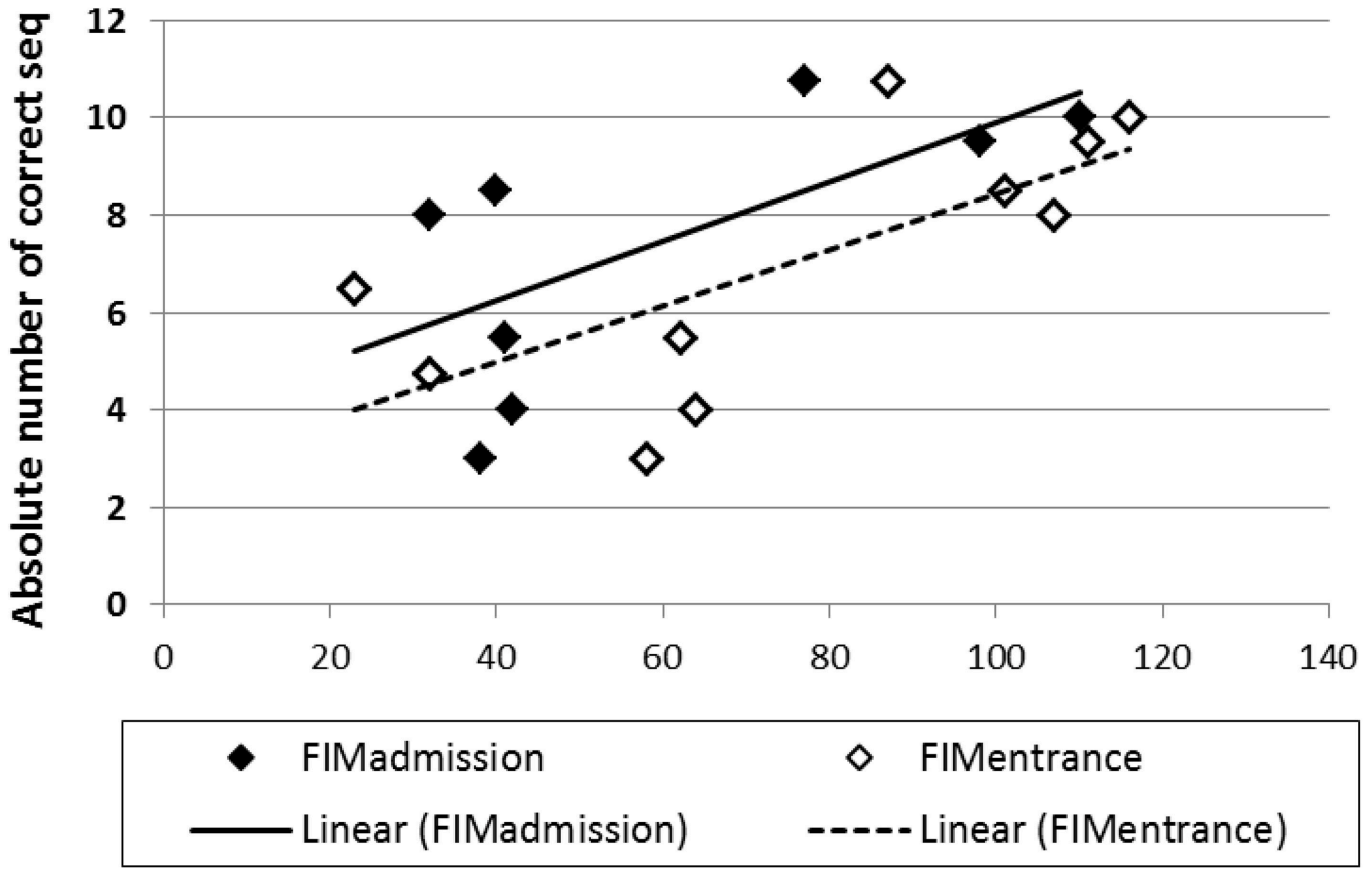
Correlations between performance speed in the initial pre-test (Test 1) and total FIM scores at admission (filled diamonds) and study entrance time points (open diamonds), x axis.

For the Trained TBI group, independent Pearson correlation tests showed that there was a significant correlation between performance speed in the initial pre-test (Test 1) and total, motor and cognitive FIM scores on arrival to rehabilitation (*R* = 0.689, *p* = 0.02; *R* = 0.681, *p* = 0.03; *R* = 0.616, *p* = 0.05; respectively) as well as a significant correlation between performance speed in Test 1 and the total, motor and cognitive FIM scores on entering the study (*R* = 0.711, *p* = 0.02; *R* = 0.707, *p* = 0.02; *R* = 0.628, *p* = 0.05, respectively; Figure 7). In addition, despite the small number of participants in the current study there was a marginally significant correlation also between the total gains attained by the end of the study period (Test 10) and the total FIM scores on entering the study (*R* = 0.807, *p* = 0.089). No significant correlations were found between performance speed at the initial pre-test (Test 1) and the time from injury, age or level of education. Given the small number of participants and some missing data the power of the following analyses was very limited; no significant correlations were found between performance speed at the initial pre-test (Test 1) and measures of cognitive abilities (LOTCA, BADS, RBMT; Table 1) or the scores obtained for upper limb motor function in the MFT or the Fugl-Meyer (Table 1). Also, no significant correlations were found between the score in the memory test (RBMT) or the MFT or the Fugl-Meyer and the individual gains in performance speed across the study period (gains from Test 1 to Test 10).

## Discussion

Severe impairments of memory and a marked reduction in the ability to learn after moderate-severe TBI are common; constituting a leading cause of disability (Vakil, 2005). The aims of the current study were: (i) to investigate the potential for procedural (“how to”) motor learning and specifically the ability to retain practice-dependent performance gains in moderate-severe patients with TBI, a few months after injury; (ii) to compare the initial level of motor performance and the course of learning of patients after TBI to those of healthy adults, in an identical protocol (adapted for patients) of the FOS task. A control group of TBI participants who were tested before and after a 6 weeks period corresponding to the study interval of the Trained TBI group, but without training afforded was included in order to differentiate between the specific effects of training and spontaneous recovery. Altogether the current results indicate that all three phases of skill acquisition, as previously observed in healthy young adults (Karni et al., 1998), can also be delineated in patients with TBI. However, the time-course of skill acquisition was atypical. Patients with TBI were much slower, compared to healthy controls, in executing the FOS at baseline and the average gain over the study period was only an additional 5.2 correct sequences (in 30 s test blocks) compared to about 12 correct sequences in healthy controls, after a similar multi-session practice protocol. Nevertheless, normalized to baseline performance, the gains attained in the Trained TBI group were comparable to those attained by the Trained Healthy group as a result of a single session of training, including across an overnight memory consolidation phase and an interval spanning a few days. The main difference in the rate of learning between the two groups, Trained TBI and Trained Healthy emerged only when training was intensified during the 2nd week of the study protocol. However, even following this second week the Trained TBI group showed very effective long-term retention, as reflected in their performance after a month long interval of no training on the task. A small but significant improvement in FOS task performance was found also by the non-trained, Control TBI group over a period of 6 weeks, but these gains were significantly smaller than those attained by the Trained TBI group. Thus, the result suggest that the while part of the gains observed in the trained group may be attributed to spontaneous recovery processes, there is clear evidence for training related gains that moreover were well maintained in memory.

The overall lower gains attained by the trained TBI participants compared with trained healthy controls can be attributed, to a large part, to the fact that initial performance levels in the TBI group were markedly reduced; patients with TBI were much slower than the healthy controls (though not less accurate). Indeed the speed attained at baseline in the execution of the FOS task by the Patients with TBI, though younger on average by about 3 decades and more, was on the order of that reported for healthy elderly individuals in a similar test condition (Korman et al., 2015). However, as the normalized data clearly indicate (Figure 5) despite the similarity in the rate of improvement between patients and healthy controls during the 1st week of the study, the rate of improvement on the task was slowed down in the TBI group during the 2nd week, when sessions of training were afforded on a daily basis. A paradoxical time-course of changes in performance evolved: the patients continued to improve between-sessions but unlike the healthy control subjects, patients showed significant losses in performance during the sessions (Figures 6B,C). In the healthy adults as well most of the gains in performance occurred between-sessions, gains that were ascribed to the engagement of procedural memory consolidation processes (Karni, 1996; Korman et al., 2003). However, even in the current protocol with its much slower pace of practice during the training sessions (potentially increasing the tediousness of the experience) and in line with previous studies (Korman et al., 2003) no within-session losses were found in the healthy controls' performance. Altogether, the results suggest that basic mechanisms of plasticity necessary for movement sequence learning, and its consolidation into long-term “how to” memory are preserved in moderate-severe patients with TBI, even in individuals with low functional baseline performance.

Korman et al. (2007) found, in healthy individuals, that the “offline” overnight improvements in performance, following a single training session, were on the order of the contribution of within-session gains. The patients with TBI in the current study, showed a pattern similar to that of the healthy controls (Figure 3B) of both within-session and between-session, consolidation phase, gains, although the high between-individuals variance and the small number of participants resulted in only marginally significant changes. Note that slower evolving “offline” overnight gains were reported in young women with ADHD after single-session training in the FOS task and ascribed to atypical, slower and thus perhaps more selective procedural memory consolidation processes (Adi-Japha et al., 2011). The current results suggest the possibility that procedural memory consolidation processes as expressed in overnight gains in performance may be evolving more slowly in some, but not all, patients with TBI and thus a clear, step-wise, increment in the initial 24 h post-training cannot be observed in the group averaged performance. Nevertheless, all of the participants of the current study showed “offline” gains in performance, following a single training session, when a few additional days were afforded for consolidation.

In both young (Korman et al., 2007) and older (Korman et al., 2015) healthy adults an interval of sleep has been implicated as a necessary factor in advancing procedural memory processes and the expression of delayed, “offline” gains in the FOS learning task. An association between sleep disturbances and motor learning has been demonstrated in patients with obstructive sleep apnea, with marked impairment in consolidation (Landry et al., 2014). Sleep quality was not assessed in the current study, although disturbances in sleep quality and architecture (Ponsford et al., 2013) may have contributed to the hypothesized slowed consolidation processes. Several studies investigated sleep after TBI with mixed results. Atypical sleep architecture in comparison with healthy controls was found in mild patients with TBI (Schreiber et al., 2008) and excessive daytime somnolence was related to changes in sleep and reduced sleep efficiency after TBI (Verma et al., 2007). Others have failed to demonstrate specific disturbances in sleep architecture in this population (Parsons et al., 1997). The current results show that patients with moderate-severe TBI are able to retain gains in performance by 24 h post-training, and importantly most individuals actually show small additional improvement in this interval. As well, in patients with TBI, between-session consolidation gains, following intensive trained sessions during the 2nd week of training, were comparable (in relative, but not absolute terms) to those of healthy controls. This suggests that sleep-dependent neuroplasticity mechanisms are at least partially preserved in TBI. It may also be the case that in some individuals with TBI, multiple sleep intervals may be needed to fully express the consolidation phase gains.

Overnight improvement occurred also during the second week of the study; however, the magnitude of each of these improvements was too small to be of statistical significance. The total gain in performance after four consecutive training sessions, however, was significant, expressing, the contribution of the between-sessions recovery and the additional gains reflecting consolidation phases (Korman et al., 2003). Thus, the overall general pattern, in the Trained TBI group, over the 2nd week of the study was one of diminished performance at the end of the daily training session and a small gain after each night's sleep. The reduction in performance speed observed immediately after each training episode may be the result of several possible factors pertaining to the medical condition; possible factors include cognitive fatigue, physical fatigue, activity related increases in pain and even an overall diminished motor ability. The overall novelty and a possibly higher level of engagement in the first training session may have offset many of these factors, resulting in both within-session and subsequent between-session gains, i.e., making the first session a more effective training experience while subsequent sessions may have become increasingly tedious. A similar notion has been suggested in the context of the length of the training session in individuals with ADHD, although in the ADHD group the effect was expressed as a reduction in accuracy (Fox et al., 2016) while in the patients enrolled in the current study speed rather than accuracy of performance was affected. The within-session reduction in performance speed is not likely to be the result of the motor impairment per-se. Firstly, there was no correlation between the gain on the FOS paradigm and upper limb function scores (MFT, Fugl-Meyer) and upper limb function scores were quite high in the TBI groups tested in the current study. Second, the pattern of improvement did not differ between TBI participants using a paretic or a motor intact upper limb. Pain in relation to the execution of the FOS task, upper extremity or finger movements was not reported by the participants, although an increase in discomfort may have had its effect toward the end of the sessions. Sleep disruptions are common in patients with TBI (Ponsford et al., 2013) and may have contributed to increased motor fatigue during the more intensive 2nd week of training.

The reduction in performance within the training session, found in the TBI group, may be related to cognitive “fatigue” including reductions in the ability to maintain attention as well as effort-reward processing. Cognitive fatigue is a process of progressive depletion of cognitive resources during sustained cognitive demands, independently of sleepiness (Roy et al., 2013). The likelihood and severity of this state may increase after TBI (van Zomeren and van den Burg, 1985; Johansson et al., 2014) and the level of fatigue may not correlate with severity or time of injury (Belmont et al., 2006). Damage to cortico-striatal pathways is a frequent finding after TBI. Cognitive fatigue might arise due to the failure of non-motor functions of the cortico-striatal system such as effort–reward processing (Dobryakova et al., 2013) and reward guided behavior (Chaudhuri and Behan, 2000; Boksem and Tops, 2008). Cognitive fatigue has also been related to reductions in goal directed attention, leading even in healthy subjects to performance in a stimulus driven fashion (Boksem et al., 2005). In a follow-up study we have conducted, increased levels of attention impairment were correlated with reduced improvements on the FOS task in participants with TBI (Stern et al, personal communication). Thus, given deficiencies in reward guided behavior and attentional capacity, practice on a daily basis may be too intensive for patients recovering from TBI, in line with the theories of cognitive fatigue (Dobryakova et al., 2013; Johansson et al., 2014). Taxing limited executive and attention capacities may lead to a reduced ability to gain from the training experience (Mathias and Wheaton, 2007). There is a need for optimizing the opportunities for rest and sleep in relation to the training protocol (Korman et al., 2015), as even in healthy adults, intensive training protocols may cause reduced performance post-training, with sleep intervals required for recovery (Mednick et al., 2008).

In addition to the negative effect of overly intensive practice on limited executive and attention capacities of patients with moderate-severe TBI (Mathias and Wheaton, 2007), the brain injury may impose specific constraints on synaptic plasticity (Albensi and Janigro, 2003; Nudo, 2013). Nevertheless, the current results indicate that even low functioning patients with TBI, including those with moderate-severe explicit memory deficits, were able to show effective motor learning in the sub-acute phase of recovery, and specifically to consolidate and well-retain skill, with no speed-accuracy tradeoff, in the performance of a complex movement sequence despite a marked reduction (compared to healthy individuals) in baseline performance measures. The study was not powered to assess the effect of the lesion site(s) on motor learning. The sample size was small and the method used to quantify the lesions was based on CT data rather than on MRI. Previous studies have underscored the contribution of specific cortical and sub-cortical regions to motor learning and “offline” motor memory consolidation in the FOS paradigm (Doyon and Benali, 2005; Debas et al., 2010; Albouy et al., 2013, 2016). Although the patients who participated in the current study had no evidence of direct damage to any area vital to this type of motor learning, disordered connectivity resulting from damage to white matter tracts may have contributed to the atypical time-course of learning and specifically the lag in gaining skill that occurred when training was given on a daily basis. Nevertheless, even in conditions apparently not well-optimized for training patients with TBI, the current results indicate extant procedural memory consolidation processes.

No correlation was found between motor skill learning, as reflected in initial task performance or in the overall gains in speed during the 2 weeks of the study, and the severity of injury, the time from injury to study enrollment or the Fugl-Meyer scores. Furthermore, no correlation was found between the initial performance level in the task or the gains in performance attained during the study and measures of cognitive abilities, including explicit memory as reflected in the RBMT. Nevertheless, even given the small number of participants in the current study there was a significant correlation between the initial performance level on the FOS task (at Test 1) and a trend toward significant correlation of the total gains attained by the end of the study period and the FIM scores on entering the study. Thus, FIM measures should be considered as a possible predictor of performance and training outcome (Shelton et al., 2001). No general conclusions can be drawn from the negative correlation analyses concerning the Fugl-Meyer and one can only make the assertion that individuals with Fugl-Meyer scores ranging between 40 and 60 (mean 57) retain a potential for learning and retention of a novel skill.

We acknowledge that our results and conclusions are based on a small sample of participants with high inter-individual differences in demographic and clinical parameters. Sex differences in motor performance and motor learning, for example, were reported in healthy participants (Dorfberger et al., 2009); higher TBI rate is associated with males (Peeters et al., 2015). The results of the current study should be taken as first, exploratory examination of the characteristics of motor skill learning time-course following multi-session training in a group of sub-acute patients with TBI. Further studies are needed to specifically address contribution of demographic and clinical parameters on the time-course of skill acquisition in patients with TBI.

In conclusion, patients in the sub-acute phase of moderate-severe TBI retain the ability to consolidate novel movement sequences and generate motor skill. Our results show that patients with TBI can express immediate as well as delayed “offline” gains in performance, the latter indicative of preserved procedural memory consolidation processes. The notion of preserved procedural memory consolidation processes in TBI is further supported by the finding that all of the patients in the current, albeit small, study, exhibited robust long-term retention. Nevertheless, the time-course of skill acquisition was atypical–most of the gains in performance evolving between-sessions and offset by losses in speed immediately after the practice sessions - and the overall gains in performance were smaller. The results also suggest that: (i) in some patients with TBI memory consolidation processes may be completed more slowly than in typical healthy adults; (ii) in intensive multi-session training protocols the patients with TBI are prone to lag behind healthy peers in terms of learning and mastering new skills, presumably due to cognitive fatigue; (iii) practice protocols and practice schedules may need to be optimized in order to better engage the potential for long-term plasticity in patients with TBI. The characterization of the neuro-behavioral constraints on motor learning after brain injury may enable caregivers to test and optimize treatment protocols, by addressing parameters that have been shown to be critical in laboratory models of skill acquisition, such as the structure and scheduling of the motor rehabilitation intervention sessions, and the use of dedicated sleep intervals between interventions.

## Author contributions

MK and SS made equal contributions. MK, SS, AK, and YS conceived and designed the experiments. KC, RM-H, and IL collected the data. KC, RM-H, IL, and CG analyzed the raw data. MK made the statistical analysis and interpretation of the data. SS, MK, AK, OK, and YS wrote the article.

### Conflict of interest statement

The authors declare that the research was conducted in the absence of any commercial or financial relationships that could be construed as a potential conflict of interest. The reviewer GB and handling Editor declared their shared affiliation, and the handling Editor states that the process nevertheless met the standards of a fair and objective review.
